# Sex hormones and allergies: exploring the gender differences in immune responses

**DOI:** 10.3389/falgy.2024.1483919

**Published:** 2025-01-07

**Authors:** Jesús Alberto Gutiérrez-Brito, José Álvaro Lomelí-Nieto, José Francisco Muñoz-Valle, Edith Oregon-Romero, Jazz Alan Corona-Angeles, Jorge Hernández-Bello

**Affiliations:** Research Institute of Biomedical Sciences, University Center of Health Sciences, University of Guadalajara, Guadalajara, Mexico

**Keywords:** allergies, sex hormones, estrogens, glucocorticoids, prolactin

## Abstract

Allergies are closely associated with sex-related hormonal variations that influence immune function, leading to distinct symptom profiles. Similar sex-based differences are observed in other immune disorders, such as autoimmune diseases. In allergies, women exhibit a higher prevalence of atopic conditions, such as allergic asthma and eczema, in comparison to men. However, age-related changes play a significant role because men have a higher incidence of allergies until puberty, and then comes a switch ratio of prevalence and severity in women. Investigations into the mechanisms of how the hormones influence the development of these diseases are crucial to understanding the molecular, cellular, and pathological aspects. Sex hormones control the reproductive system and have several immuno-modulatory effects affecting immune cells, including T and B cell development, antibody production, lymphoid organ size, and lymphocyte death. Moreover, studies have suggested that female sex hormones amplify memory immune responses, which may lead to an excessive immune response impacting the pathogenesis, airway hyperresponsiveness, inflammation of airways, and mucus production of allergic diseases. The evidence suggests that estrogens enhance immune humoral responses, autoimmunity, mast cell reactivity, and delayed IV allergic reactions, while androgens, progesterone, and glucocorticoids suppress them. This review explores the relationship between sex hormones and allergies, including epidemiological data, experimental findings, and insights from animal models. We discuss the general properties of these hormones, their effects on allergic processes, and clinical observations and therapeutic results. Finally, we describe hypersensitivity reactions to these hormones.

## 1 Introduction

Allergies are the most common chronic inflammatory disorders worldwide; triggered by immune reactions to specific allergens, which can be proteins or glycoproteins and vary widely in nature and origin (e.g., pollen, dust mite dander, food) (1). Vary allergic conditions (e.g., urticaria, asthma, atopic dermatitis, rhinoconjunctivitis, allergies to food or drugs, and anaphylaxis) share common pathogenic mechanisms, though the precise pathways remain not entirely clear (2). Additionally, epidemiological data indicate notable differences in the incidence, prevalence, and severity of allergies between sexes (3).

Globally, the prevalence of allergies is rising, with documented cases indicating that 400 million people suffer from rhinitis, 300 million from asthma, between 200 and 250 million from food allergies, and approximately 10% of the world population from drug allergies (4). Although the precise mechanisms underlying this rapid increase in prevalence are unknown, many factors can influence allergic responses; emerging evidence suggests that genetics, environment, microbiota, diet, and sex-related factors may play a significant role (5).

Allergies are generally mediated by expanding populations of helper T cells subtype 2 (Th2), which produce type 2 cytokines such as interleukin IL-4, IL-5, IL-9, and IL-13. These cytokines influence the pathology of allergies; IL-4 helps B cells differentiate into IgE-producing plasma cells, triggering an immediate allergic reaction after binding to high-affinity IgE Fc*ε*RI receptor on mast cells (6). It also promotes Th2 cell differentiation. IL-5 induces differentiation, recruitment, and activation of eosinophils (7), while IL-13 increases mucus production in the bronchial epithelium and participates in B cell activation (8).

**Figure 1:**
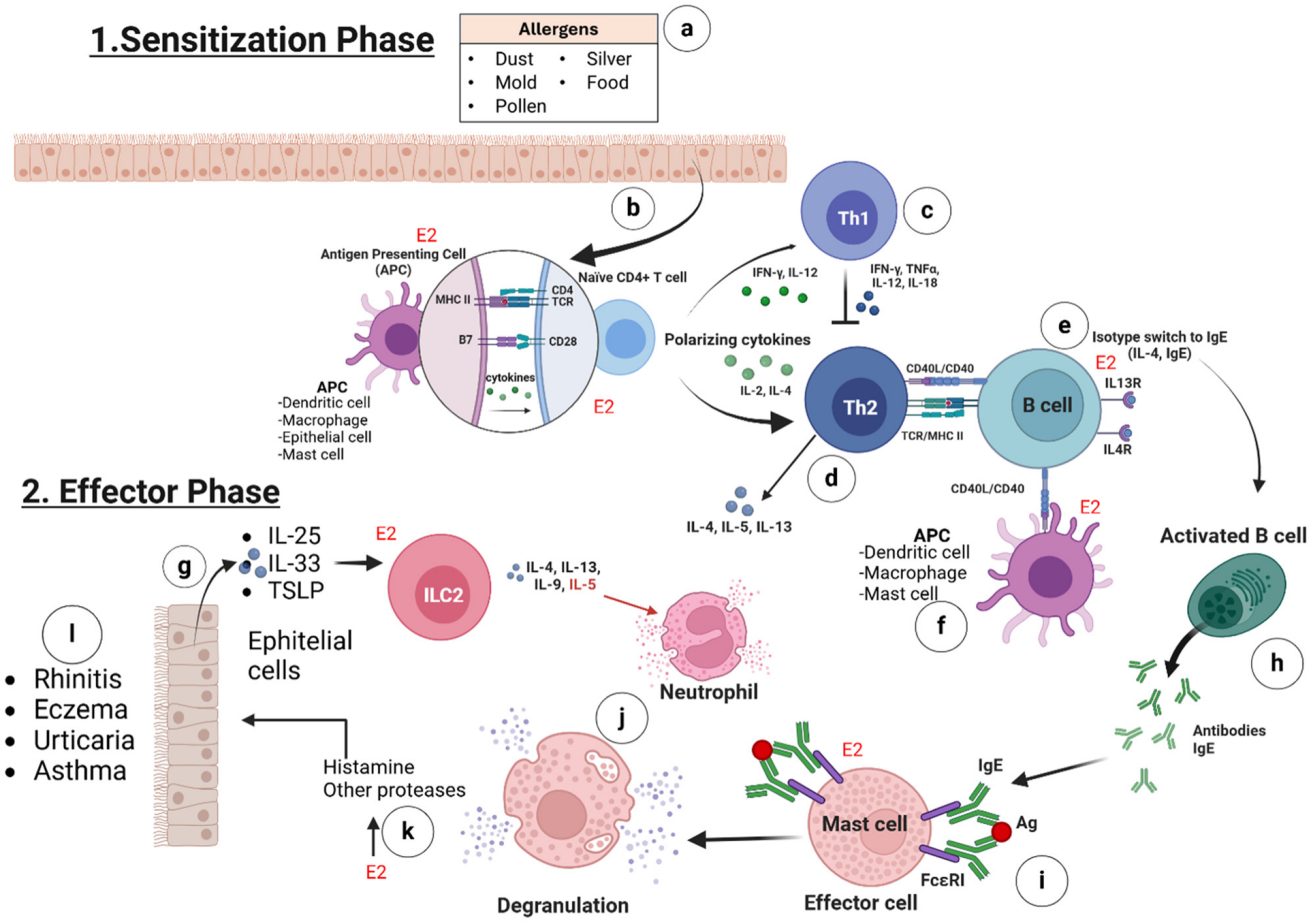
The intricate stages of the allergic response. 1. Sensitization phase involves **(a)** the contact of the allergens (e.g., dust, mold, pollen, silver and food) with the body's tissues and starts the allergic process; **(b)** estrogens (*via* ERα, ERβ, GPER, and E2) promote the synthesis of MHC-II and B7 receptors on the membranes of antigen-presenting cells (APCs) and enhance cytokine production to activate naïve CD4+ T cells; **(c)** in the absence of estrogens, naïve CD4+ T cells differentiate into Th1 cells, producing IL-12, IL-18, TNF-α, and IFN-γ, which inhibit Th2 cell differentiation; **(d)** in the presence of E2, naïve CD4+ T cells differentiate into Th2 cells, producing IL-4, IL-5, IL-9, IL-13, or IL-10, which subsequently activate B cells; **(e)** B cells are activated by IL-4, IL-5, IL-9, and IL-13, and in the presence of E2, they express more CD40 receptor on their membranes; **(f)** APCs (e.g., activated mast cells) may bind to B cells through the CD40l/CD40 interaction and this lead also the activation of B cells. 2. The effector phase involves **(g)** the production of IL-25, IL-33, and TSLP in response to tissue damage or stress from allergen contact, which activates ILC2 cells. These cells then produce IL-5, IL-9, IL-13, and IL-4; IL-5 plays a key role in eosinophil recruitment and activation, while all are crucial for B cell activation; **(h)** the isotype switching of activated B cells to plasma cells results in the production of specific IgE antibodies; **(i)** estrogens promote the expression of Fc*ε*RI on mast cells, which is a specific receptor for IgE binding; **(j)** the binding of the antigen, in the reexposure to the allergen, to IgE-Fc*ε*RI on the mast cells, triggers the degranulation of some proteases and, primarily, histamine; **(k)** the presence of E2 enhances degranulation by increasing expression of Fc*ε*RI on mast cells; **(l)** the inflammatory enzymes and histamine can lead to symptoms such as rhinitis, eczema, urticaria, or asthma, depending on tissue involvement. (Created with BioRender).

The allergic response can be divided into two phases: sensitization and effector. During the sensitization phase, allergens can break through the body's protective mucosal barrier and be taken in by an antigen-presenting cell (APC). The APC degrades the allergen into peptide fragments and presents them to major histocompatibility complex (MHC) class II molecules. This interaction activates CD4+ T cells or Th2 cells, which, in turn, stimulate naïve B cells to differentiate into IgE-producing plasma cells (9). Upon re-exposure, allergens bind to IgE on sensitized mast cells or basophils, inducing cross-linking of IgE-Fc*ε*RI complexes, which triggers cell activation and degranulation. During the effector phase, degranulation results in the release of histamine and proinflammatory cytokines from these cells. The release of these mediators produces allergy symptoms in various organ systems and tissues, including the alveoli (Figure 1). These symptoms include upregulating inflammation, eosinophilia, smooth muscle contraction, excessive mucus secretion, vasodilation, and tissue damage; thus, persistent exposure to allergens causes the disease to become chronic (10).

Interestingly, hormonal variations have been identified to affect the immune system. After puberty, males are believed to maintain higher or similar total serum IgE and allergen-specific IgE levels compared to females. As adults, both sexes experience a decrease in IgE levels. In addition to age-related changes, menstrual cycles and pregnancy can also influence IgE levels, suggesting the role of estrogens in their regulation (11).

Clinical data supports the role of sex in the sensitization process, indicating that women may be more prone to allergies than men, including conditions such as urticaria, anaphylaxis, food allergy, and asthma (12). Additionally, clinical investigations and longitudinal studies have highlighted the higher prevalence of systemic allergic reactions in women, who are more susceptible than men to both idiopathic allergic reactions and those induced by food, drugs, and radiocontrast agents (13, 14).

Specific associations have been reported between pregnancy, hormonal therapies, and the severity of allergic reactions (15). Therefore, differences among sexes can significantly affect allergy prevention, detection, diagnosis, and treatment (16). Immunological sexual dimorphism can cause discrepancies in allergic responses in men and women because of a dynamic and complex combination of hyperreactivity, deregulated immune response, chronic inflammation, and tissue remodeling in affected organs (17).

Regarding asthma, global reports indicate variations between sexes. It is observed that men have a higher incidence of asthma until puberty. Still, after puberty, there is a switch in the ratio, and the prevalence and severity of asthma increase in women (3, 18). Furthermore, approximately 30 to 40% of asthmatic women report cyclical changes in the disease during pre and perimenstrual phases, leading to a reduction in lung function, severe asthma attacks, needing more bursts of corticosteroid, and having to be hospitalized (2, 11). Moreover, hormonal fluctuations during pregnancy and menopause may be responsible for exacerbations of asthma in women (19).

In addition to asthma, Eczema is more common among boys during childhood, but girls are more likely to have it after puberty. This indicates a switch from male to female predominance by early adulthood (20). Another example is what happened in Vernal keratoconjunctivitis, a severe ocular allergy that mainly affects boys but almost disappears after puberty (21). Even in the cases of peanut allergy, females are more affected than males (22). This could have occurred because androgens produced in more significant amounts in men after puberty generally suppress the responsiveness of immune cells (23). In this sense, changes in the sex hormones, such as those occurring at puberty, may influence the development of allergic diseases.

Although the effect of sex hormones on immune cell function, innate immune sensing in the airways, development, T-cell activation, and B-cell responses have been extensively studied, the mechanisms by which sex hormones specifically influence the sensitization process in individuals of different age groups, such as young people, adolescents, and adults remain unclear.

Age-related and sex-based differences in allergic disease prevalence highlight the significant roles of biological sex and gender-related factors in allergy development and progression. Boys tend to have a higher incidence of allergic conditions in childhood, but this trend reverses at puberty, leading to a higher prevalence in women throughout adulthood. This shift likely arises from complex interactions involving sex hormones, genetic and epigenetic regulation, lifestyle factors, microbiota diversity, and environmental exposures. Such insights emphasize the need for further studies to better understand the underlying mechanisms and the influence of gender-specific factors in the sensitization and development of allergic diseases across different life stages (24).

## 2 Sex hormones and the immune system

The term sex hormone typically refers to sex steroid hormones, which are derived from cholesterol metabolism (25). However, other non-steroidal hormones, like prolactin (PRL), which plays a vital role associated with sex, are not usually considered within this category (26, 27).

Although sex hormones have well-known effects on sexual differentiation and reproduction, they also influence the immune system and cause sexual dimorphism in the immune response (28). Thus, women often develop more robust immune responses than men, making them more resistant to certain infections (29).

However, increased immunity has significant drawbacks, particularly the high prevalence of autoimmune and allergic diseases among women. Over 80% of patients with antibody-mediated autoimmune conditions, such as systemic lupus erythematosus, Sjögren's syndrome, and Hashimoto's thyroiditis, are women. This suggests that the immunological mechanisms underlying sex differences in infectious diseases and acquired immunity to pathogens may also contribute to the higher rates of autoimmune and allergic diseases observed in women (5). These findings suggest that hormonal differences between sexes may modulate the normal and dysfunctional regulation of the immune system.

Allergic inflammation of the airways, characterized by the presence of the Th2 cytokines profile (19), is stronger in women, probably because estrogens could enhance it (30). In contrast to the drop in asthma incidence observed in and around puberty in males, the increased number of remissions observed intensely suggests the protective action of male sex hormones (31).

Some asthmatic patients present a reduced (or no) Th2-mediated airway profile; however, they present an increase in neutrophils driven by airway inflammation mediated by Th1 or Th17 cytokines profiles. These cases may respond to different causes, e.g., non-allergic asthma, corticosteroid resistance, and genetic or environmental factors, such as high levels of hormones like testosterone (32, 33).

Animal studies suggest that pregnancy is associated with cytokine polarization towards a Th2 profile, with early increases in several cytokines followed by a decrease in Th1 cytokines, such as IL-2 and Interferon gamma (IFN-γ), and an increase in Th2-like cytokines, such as IL-4, as pregnancy progresses. Thus, the physiological increase in cortisol, progesterone, estradiol, and testosterone concentrations observed during the third trimester of pregnancy could be involved in Th2-like cytokine polarization (34).

These findings suggest that sex hormones could have essential implications in immunological pathogenesis, such as asthma and hypersensitivity reactions. However, the underlying mechanisms must be fully elucidated (19, 35).

Hormones also regulate the thymus's structure and function, affecting B and T lymphocytes, mast cells, natural killer (NK) cell activity, phagocytic cells, and cytokine production (36). Additionally, they regulate type 2 innate lymphoid cells (ILC2), which are more abundant in asthmatic women than in men's peripheral blood (37).

Hormones can make these effects by binding to their receptors, triggering intracellular signaling cascades that can induce the expression of immunomodulatory genes in the pathophysiology of allergies (38).

The sex hormone receptors could be present on the surface of immune cells like transmembrane proteins or, more classically, in the cytoplasm. These receptors trigger specific cellular-type responses; for example, they can modulate antigen presentation mediated by dendritic cells, mast cells, or monocytes/macrophages, which is why they are crucial to determining the type of adaptive immune response under normal or pathological conditions (39).

Scientific evidence suggests that female sex hormones act as enhancers of autoimmunity, mast cell reactivity, and delayed-type IV allergic reactions. At the same time, male hormones and glucocorticoids may have an immunosuppressive effect (40, 41).

The following sections summarize the specific effects of sex steroid hormones and PRL on allergic reactions in humans and some animal models.

## 3 Steroid sex hormones

Sex hormones and corticosteroids are complex organic molecules of four rings produced in several organs, such as the adrenal glands, gonads, placenta, and adipose tissue. They all originate from cholesterol through the same steroidogenic pathway. Sex hormones include androgens (such as testosterone and androstenedione), estrogens (like estradiol, estrone, and estriol), and progesterone (42).

Other non-steroid sexual hormones, like follicle-stimulating hormone (FSH) and luteinizing hormone (LH), have low levels during menopause and are associated with allergies, such as asthma (35). Additionally, PRL is involved in allergic lung inflammation (43).

The steroid hormones function as endogenous endocrine hormones, exerting physiological effects in cells by binding to and activating specific intracellular proteins called nuclear receptors (NRs). Class I NRs include estrogen receptors alpha (ERα) and beta (ERβ), androgen receptor (AR), glucocorticoid receptor (GR), progesterone receptor (PR), and mineralocorticoid receptor (MR) (44).

Hormones readily penetrate the cell membrane to the cytosol; NRs are located mainly in an inactive state, forming an inactive complex with heat shock proteins (HSP) (27). When a steroid ligand binds to its receptor, it dimerizes, then translocate to the nucleus and binds to specific palindromic sites called hormone response elements (HRE) located in the promoters of target genes; NRs can activate or repress their transcription, which means that they act as transcription factors (30, 31, 36).

The effects mediated by hormones are also due to their ability to regulate gene expression without direct binding to DNA but rather through protein interactions with other transcription factors that bind to DNA, including nuclear factor -κB (NF- κB), specific protein 1 (Sp1), CCAAT-enhancer-binding protein β (C/EBP β), or Fos/Jun AP-1 complex (activator protein-1); which are all involved in the synthesis of proinflammatory cytokines by innate immune cells (45, 46).

### 3.1 Estrogens

Over the years, several studies have investigated the immunological mechanisms by which estrogens influence and alter immune cells, potentially contributing to the development of autoimmune diseases and allergies. These pathologies share common pathogenic mechanisms, and estrogens remain among the most studied hormones regarding their impact on allergic disease pathology.

Estrogens control both sexes' critical processes, such as glucose and lipid metabolism, brain function, growth, and cell activation (47). Moreover, they play an active role in producing proinflammatory cytokines and have different effects throughout the menstrual cycle, pregnancy, and menopause (48, 49).

In adulthood, there are three physiological forms of estrogens: estrone (E1), estradiol (E2), and estriol (E3). Studies have described that E2 is the principal and potent circulating estrogen, with various effects on the immune system cells, including its ability to modify the differentiation, maturation, and effector functions of conventional dendritic cells, which play a critical role in activating and polarizing the adaptive immune responses (50).

E2 is also attributed to immunomodulatory effects (51), suppressing the effector function of T lymphocytes and increasing Th2 cell function and subsequent antibody production (52–55). In addition, E2 has been associated with delayed-type hypersensitivity responses (54) and relatively higher antibody responses in women after vaccination, except IgG titers (56).

Estrogens can also control immune tolerance through epigenetic and transcriptional mechanisms. They suppress DNA methyltransferase 1 (DNMT1), causing hypomethylation in CD4+ T cells, increasing activation. Also, an imbalance in histone acetylation profiles has been observed in Peripheral blood mononuclear cells (PBMCs). Estrogen also impacts transcription factors, like AIRE, FOXP3, and RORγT, which are crucial for immune tolerance control (57).

Estrogens mediate signaling by binding to their receptors, which belong to two families: the G protein-coupled estrogen receptor (GPER) and the NRs (ERα and ERβ) (58, 59). ERα and ERβ can also be located in the cell membrane, initiating membrane-initiated steroid signaling (MISS). This allows for a faster response to estrogens compared to classical genomic signaling, in which estrogens pass through the membrane and bind to the receptors in the cytosol (60).

GPER is a seven-transmembrane domain protein that binds specifically to E2 and is relevant in regulating non-genomic actions (not involving transcriptional components). It is encoded by the gene *GPER1* located in the human chromosome 7; the protein is expressed on the surface of the membrane and at intracellular membranes (61). This receptor mainly mediates rapid intracellular responses induced by estrogens, including activation of intracellular signaling exemplified by AKT and MAPK pathways (62–64). Indeed, GPER is expressed in non-immune cells and cells from the innate and adaptive immune system, including circulating B lymphocytes, T lymphocytes, monocytes (65), macrophages (66), neutrophils (67–69), and eosinophils (70).

On the other hand, the classical or genomic (nuclear) pathway is mediated by the ERα and ERβ. Both ERs bind the same ligands with similar affinity (60) and are encoded by *ESR1* and *ESR2* genes located on human chromosomes 6 and 14, respectively (71). Interestingly, some single nucleotide variants in the *ESR1* gene have been associated with hypersensitivity and allergies in asthmatic women (72).

**Figure 2:**
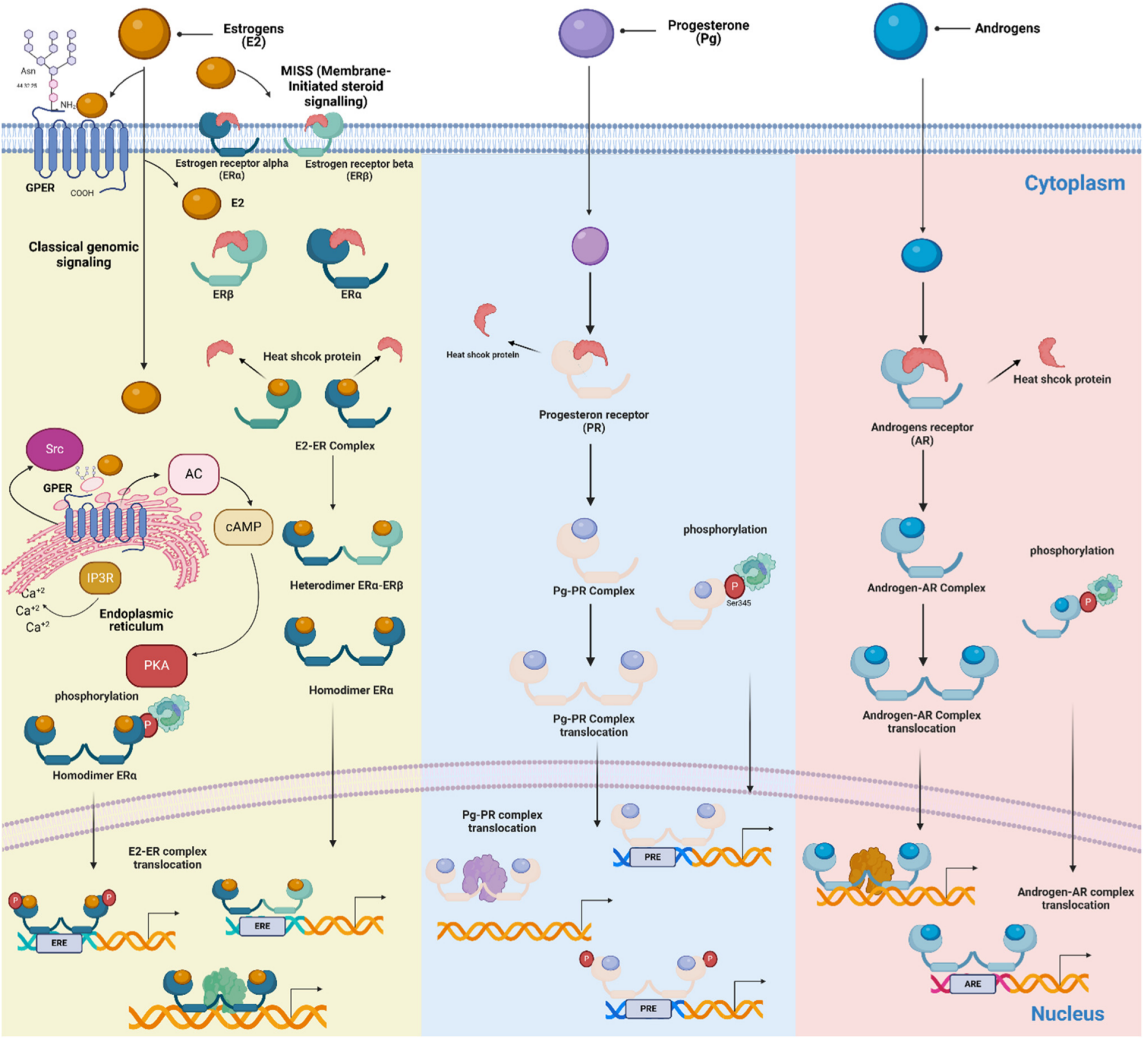
Schematic of mechanisms of steroid action on cellular responses. Genomic and non-genomic, ligand-dependent and ligand-independent, classic and non-classic receptor-mediated steroid-steroid receptor signaling pathways are shown. Membrane-Initiated Steroid Signaling (MISS) is also depicted, representing the rapid response initiated when membrane-bound estrogen receptors interact with estrogens. The receptors are found in the cytoplasm of target cells, where they are associated with large heat-shock protein-containing complexes that keep the receptors inactive. When the steroid hormone receptor binds to the ligand, it undergoes a series of changes. It releases inhibitory proteins, forms dimers, moves into the nucleus, and interacts with specific enhancers or hormone response elements near the promoters in target genes. This process of activation allows the receptors to directly interact with specific estrogen response elements on DNA or be attached to DNA through interactions with other transcription factors that are already at the enhancers. Signaling pathways like MAPK can converge on receptor-coregulator complexes, modifying their activity and enabling receptors to activate target gene transcription without a classical ligand. (Created with BioRender).

ERs can regulate cellular function through two different mechanisms: The nuclear, in which the activated ERs form homodimers or heterodimers to tightly fix chromatin directly at the estrogen-responsive element (ERE) sites or indirectly at AP1 or Sp1 sites (Figure 2). ERs can then remodel chromatin by recruiting cofactors and activating RNA polymerase II at target genes (genomic action). The second is the non-genomic mechanism; ERs on the cell membrane (mERs) initiate signals through kinases such as MAPK, PI3K, and GFR pathways (73).

The ERα and ERβ are expressed in several immune system cells, including T suppressor/cytotoxic (74), helper T, and B cells (75). The expression levels vary between cell types. For instance, T CD4+ cells and M2 macrophages express more ERα, while B cells and respiratory tract epithelial cells express more ERβ (76–78). Furthermore, the expression of both receptors has been found in mature lymphocytes, which explains the immunological changes that occur in women when estrogen is present (79, 80).

The study by Narita et al. showed the effect of E2 on cultured mast cells, where E2 stimulation enhanced IgE-induced degranulation. This indicates that estrogen stimulation can lower the threshold of allergens. The study also demonstrated that ER*α* mediates part of the degranulating activity of estrogen on mast cells (81).

The action of estrogens in the allergic sensitization phase has been studied in APCs differentiated cells, including dendritic cells, macrophages, epithelial cells, and mast cells. It has been shown that E2 in APCs increased expression of MHC-II and costimulatory molecules like B7 receptors or CD40. The expression of these receptors facilitates the allergic sensitization and activation of B cells to IgE plasma cells (82, 83).

Despite limited information on the role of GPER in allergies today, studies have shown contradictory findings. The study by Tamaki et al. showed that activation of GPER through G-1 (a specific agonist) on human eosinophils inhibits the enzymatic activity of caspase-3 and reduces spontaneous apoptosis, suggesting a potential mechanism for GPER's impact on asthma pathogenesis; thus, these findings prove a mechanism of direct interaction between estrogen and eosinophil functions (70). If female hormones enhance eosinophil function, it could signify that estrogens accelerate allergic inflammation.

In contrast, the recent study by Itoga and colleagues reported different functions of GPER. They found that when G-1 is administered to a mouse model of chronic asthma, it suppresses allergic airway inflammation. They observed that the G-1 treated group had significantly fewer peribronchial inflammatory cells, eosinophils, and lymphocytes in their bronchoalveolar lavage fluid (BALF) than the untreated group. Additionally, in splenic mononuclear cells, the percentage of Foxp3 + CD4+ Treg was significantly increased in the G-1 treated group. In conclusion, GPER could be a therapeutic target for allergic airway inflammation (84).

Furthermore, studies suggest that estrogens play an essential role in the proliferation and survival of B cells and the antibody or autoantibody response (85), which may explain the higher prevalence of autoimmune diseases in women. Estrogens can induce the expression of critical molecules in B cells, such as CD22, SHP-1, and Bcl-2, which regulate apoptosis processes through genetic programming (82). ER-mediated transcriptional regulation induces class switching to IgE and somatic hypermutations in developing B cells (85, 86). It has also been shown that estrogen and estrogenic compounds can promote IgE production in mouse splenocytes, which may worsen allergic inflammation (87). Furthermore, estrogen increases histamine secretion from human basophils, an essential mediator of allergy and inflammation in asthmatic patients (87).

Estrogens also promote the dissociation of endothelial nitric oxide synthetase (NOS), activating the nitric oxide (NO) pathway, vasodilatation, and consequently increased inflammation (87). An *in vitro* study on bronchial epithelial cells showed that treatment with 10 nM of estrogen induces NOS expression and NO production through the ERs, resulting in bronchodilation (88).

Macrophages are crucial in allergic inflammation, particularly in allergic asthma, where alveolar macrophages are central to inflammation and tissue remodeling; in this disease, IL-4/13 promotes the polarization of M1 to M2 type macrophages in the airways (89). Indeed, the study by Keselman et al. showed that women stimulated with estrogens produce a more significant amount of IL-4, promoting the polarization to M2a-type macrophages that participate in eosinophil infiltration in the alveoli, producing inflammation and greater severity (89).

Bone marrow-derived macrophages (BMMs) from ovariectomized mice serve as an *in vitro* model for studying the effect of estrogens on macrophage biology. These cells show decreased *TGFβ1* mRNA levels, which are rescued by estrogen treatment. This study highlights the role of estrogens in tissue remodeling during allergic inflammation by inducing TGFβ1 (89). In experimental models of asthma, female mice, after allergen challenge, have increased airway hyperresponsiveness, eosinophil influx, and more cytokine type 2 production (IL-4, IL-5, and IL-13) compared to males. Moreover, ILC2s produce more IL-5 and IL-13 than Th2 cells (90).

Moreover, estrogens can induce the relaxation of airway smooth muscle (ASM) (91), leading to bronchodilation and more frequent exacerbations in the presence of low E2 levels (48). Another finding is that E2 can influence the production of secretory leukocyte protease inhibitor (SLPI), which inhibits a serine protease and thus protects against tissue damage (92).

Studies by Macsali et al. and Erkoçoğlu et al. indicate that oral contraceptives containing estrogens and progestogens are associated with increased wheezing in women with asthma (93, 94). Menopause, which leads to lower estrogen levels, is also associated with worsened asthma symptoms (95), suggesting that estrogen may have a protective effect at certain levels (96). Hormone replacement therapy (HRT) can increase estrogen levels and alleviate postmenopausal symptoms (97); however, new asthma diagnoses tend to rise following HRT initiation and decrease after its discontinuation (98). These findings suggest that estrogen's effect on asthma symptoms may vary based on dosage, with both very low and high levels potentially contributing to adverse respiratory outcomes (96).

Postmenopausal women have a higher prevalence of allergic rhinitis, which has been associated with a decrease in estrogen levels and a corresponding shift in immune profile. This shift away from a Th2-dominant response may alter IgE regulation and contribute to increased susceptibility to allergic conditions (99). On the other hand, during pregnancy, fluctuating estrogen levels can influence the course of allergic rhinitis. While some women experience a worsening of pre-existing symptoms, others may develop a distinct condition known as pregnancy-induced rhinitis, which affects approximately 20% of pregnant women across all trimesters and resolves shortly after delivery (100). Despite these seemingly contradictory effects, estrogens have also been shown to have a protective role by promoting the activation of regulatory T cells, IL-10, and FOXP3, which can help alleviate symptoms of allergic rhinitis (101). This duality in the action of estrogens suggests that their influence on allergic rhinitis depends on the specific physiological context and interactions with other hormonal and immunological factors.

Finally, it is important to note that xenoestrogens and estrogenic environmental pollutants (e.g., dioxins) interact equally with ER*α* and ER*β* receptors. These contaminants could promote allergic diseases by supporting the release of histamine and an allergic reaction, thus facilitating their release (81, 102). However, recent research has revealed a unique aspect of phytoestrogens, such as secoisolariciresinol diglucoside (SDG), found abundantly in flaxseed, which undergoes metabolic conversion to exert anti-allergic properties. In an ovalbumin-induced allergic rhinitis mouse model, it was demonstrated that dietary SDG alleviated allergic rhinitis through its microbial conversion to enterodiol (ED). Notably, ED circulated primarily in the glucuronide form (EDGlu) in the blood, and deconjugation to ED aglycone occurred in the nasal passage, an activity enhanced after the induction of allergic rhinitis and mediated by β-glucuronidase. Furthermore, ED aglycone, but not EDGlu, inhibited IgE-mediated degranulation in a G protein-coupled receptor 30 (GPR30)-dependent manner. These findings provide new insights into the anti-allergic properties of phytoestrogens and their potential metabolism *in vivo*, suggesting that estrogens, including phytoestrogens, could be a novel target for therapeutic strategies against allergic rhinitis (103).

### 3.2 Progesterone

Progesterone (Pg or P4) is a steroid hormone synthesized by several organs during pregnancy, including the adrenal glands, ovaries (by the corpus luteum), testicles, brain, and placenta. It serves as a precursor for the biosynthesis of androgens, estrogens, and corticosteroids and has many metabolic and physiological functions related to the menstrual cycle, pregnancy, embryogenesis, and lactation (104, 105).

Pg has immunomodulatory and anti-inflammatory effects that inhibit glucocorticoid-mediated apoptosis of thymocytes, macrophage activity, and IFN-γ production in NK cells (84–87, 195, 196). It also reduces NO production (106) and TLR expression by macrophages (107) and promotes the differentiation of Th2 lymphocytes *in vitro* (90).

Pg exerts its effects through intracellular Pg receptors (PR). Additionally, it has rapid, non-transcriptional actions mediated by membrane Pg receptors (mPR), which are structurally distinct (108).

Studies have demonstrated that PR expression is absent in neutrophils, eosinophils, and B cells (109, 110). In contrast, PR is present in mast cells, natural killer (NK) cells, macrophages, dendritic cells, CD4+ T cells, and CD8+ T cells, though its characterization in these cells is not yet fully understood (94–98).

When Pg binds to its receptor, it triggers intracellular signaling cascades, including classical and non-classical pathways (Figure 2), leading to different effects in immune cells (197). For example, in T lymphocytes, Pg induces the release of progesterone-induced blocking factor (PIBF), which stimulates the expression of cytokines, such as IL-4, IL-5, IL-6, IL-9, IL-10, and IL-13. Meanwhile, in NK cells, Pg decreases the expression of IFN-γ and regulates their localization and proliferation (111). In contrast, the effect of Pg on mast cell degranulation is still unclear. Some studies suggest activation (112) and another inhibition effect (113).

In mouse models, Pg has shown anti-inflammatory effects (114). Furthermore, in some cases, severe premenstrual asthma exacerbation has been treated effectively with Pg, likely due to smooth muscle relaxation and regulation of microvascular permeability (115).

In another study, lower doses of progesterone were found to stimulate TNF-α production in human and mouse macrophages. In comparison, higher doses were shown to suppress cytokine release and IL-1 mRNA expression (116). These findings suggest that the hormonal profile is vital in releasing inflammatory mediators.

### 3.3 Androgens

The gonads and adrenal glands synthesize four androgens, including dihydrotestosterone (DHT), testosterone, androstenedione, and dehydroepiandrosterone (DHEA), all derived from cholesterol. DHT is considered more potent than testosterone, whereas androstenedione and DHEA have a lower potency than testosterone, with only 10% and 5%, respectively (117). In adult men, testosterone is found in the serum in higher concentrations than other androgens. At the same time, DHT is present at a concentration of approximately one-tenth of testosterone. Additionally, DHEA can undergo reversible modifications to produce DHEA-S, which can be metabolized peripherally to form testosterone (particularly in premenopausal women) and estrogens (particularly in postmenopausal women) (118).

Androgens such as testosterone and DHT primarily exhibit immunosuppressive effects, inhibiting the activities of Th1, Th2, and Th17 cells, while promoting the activity of regulatory T cells (Tregs) (119). DHT has been shown to suppress proinflammatory gene expression (120). Androgens inhibit Th1 differentiation by reducing the production of IFN-γ and the expression of T-bet in CD4+ T cells. An *in vitro* study using an androgen analog (R1881) shows that CD4+ T cells cultured under Th1-polarizing conditions in the presence of androgens produce significantly less IFN-γ compared to controls. This effect is specific to the differentiation phase, as cells differentiated without androgens but restimulated in their presence produce normal IFN-γ levels. The findings suggest that androgen impacts Th1 differentiation by limiting the early molecular events essential for this immune pathway (121).

In a model of male castrated mice with allergic rhinitis sensitized to phospholipase A, administration of testosterone reduced the production of phospholipase A-specific IgE, demonstrating the inhibitory effects of androgens on allergic rhinitis (122). Furthermore, in asthma, studies have shown that DHEA may improve lung function, reducing symptoms (123). Phase II clinical trials have demonstrated that oral administration of slow-release or nebulized DHEA to men and women with severe asthma decreases symptoms and increases lung function (123–125).

Lower levels of DHEA have been associated with increased severity of asthma, especially in relation to age. Conversely, studies have shown an inverse correlation between serum testosterone levels and the prevalence of asthma (126). Additionally, other studies demonstrate that higher testosterone levels are linked to improved lung function, as evidenced by higher forced expiratory volume in one second (FEV1) and forced vital capacity (FVC) across various racial and ethnic groups (127, 128).

Additionally, it was observed that individuals with asthma, with increased expression of the AR in their airways, tend to have better lung function and lower fractional exhaled nitric oxide (eNO) (a marker of inflammation) (129). Interestingly, when nebulized dehydroepiandrosterone 3-sulfate was administered, it improved asthma control scores for people with moderate-to-severe asthma (124).

The administration of DHEA and its conversion rate to androgens have been linked to improved responsiveness to glucocorticoids or lung function in asthma patients (124, 125, 130). However, while AR signaling does not directly decrease the production of Th2 cytokines (131), it enhances the suppressive function of regulatory T cells (Tregs), thereby reducing allergic inflammation of the respiratory pathways. This is achieved by enhancing Treg stability by limiting allergen-induced IL-33 production in epithelial cells and *ST2 expression* in Treg cells (132). These findings highlight the clinical relevance of androgen- and its receptor-mediated signaling in attenuating airway inflammation in asthma.

Androgens also affect ILC2 cells, which are crucial in producing IL-5 and IL-13. Specifically, androgens attenuate the differentiation of ILC2 and decrease their numbers within the lung (37, 133).

In a murine model of allergic asthma, these hormones showed an effect by reducing neutrophilic inflammation (134). Furthermore, in a model of allergic rhinitis, castrated males had higher levels of antigen-specific IgE compared to a control group that underwent a sham operation. Interestingly, when the castrated mice were treated with androgens, the levels of antigen IgE decreased significantly (135). This finding underscores the role of androgens in modulating different kinds of responses. Therefore, in male guinea pig asthmatic models, androgens positively affect through different mechanisms, including the relaxation of ASM via an epithelial pathway that relies on nitric oxide (NO), demonstrated by dehydroepiandrosterone (136).

The AR acts as a transcription factor requiring ligand binding to activate gene expression. The human AR is a 110 kD protein composed of 919 amino acids (137, 138). The expression of the AR has a much more restricted expression pattern in the body; it has been found in several immune cells, such as neutrophils, mast cells, macrophages, B cells, and T cells (134, 139), and such immune cells found in bone marrow, thymus, and spleen (120).

The androgen signals and ARs are responsible for controlling the development and function of both male and female reproductive systems (140). However, AR signaling has a different effect on the immune response compared to ERs. By inhibiting the immune response, AR signaling may explain why males are more susceptible to common pathogens (120).

Studies using immune cell-specific AR knockout mouse models have demonstrated that androgens and their receptors play a significant role in innate and adaptive immunity. For instance, the binding of androgen/AR is essential for proper neutrophil generation and function and for regulating wound healing processes by recruiting macrophages and promoting proinflammatory cytokine production. Additionally, this binding suppresses T and B cell development and activation in adaptive immunity (134). Therefore, the role of androgens in allergies should be further explored using these approaches in humans.

According to Becerra-Díaz et al. (141) found that androgens increase the polarization of alveolar macrophages (AM) to an M2 phenotype via AR, upregulating the expression of the genes *Chil3l3*, *Retnla*, and *Arg1* (141).

In summary, androgens have immunosuppressive effects and may critically modulate immune responses in specific pathological conditions, presenting promising avenues for future research and treatment.

## 4 Glucocorticoids

Glucocorticosteroids (GCs), also known as corticosteroids or steroids, are natural regulators that play a vital role in regulating various biological processes such as the hypothalamic-pituitary-adrenal axis, immunity, and energy metabolism to maintain balance within the body. Cortisol, the primary glucocorticoid synthesized and secreted by the adrenal cortex, interacts with the GC receptor (GR) to regulate multiple human signaling pathways (142). Synthetic GCs are immunosuppressive drugs for treating immune-related disorders, including allergies (143). The most common treatments for asthma are inhaled glucocorticoids for suppression of inflammatory gene expression and β2-adrenergic receptor agonists for inhibition of bronchoconstriction (144).

Synthetic GCs bind to its cognate intracellular receptor GR with higher affinity than endogenous glucocorticoids, which means synthetic glucocorticoids are more potent immunoregulators than cortisol because they are not subject to endogenous inhibitors of cortisol activity (143).

**Figure 3:**
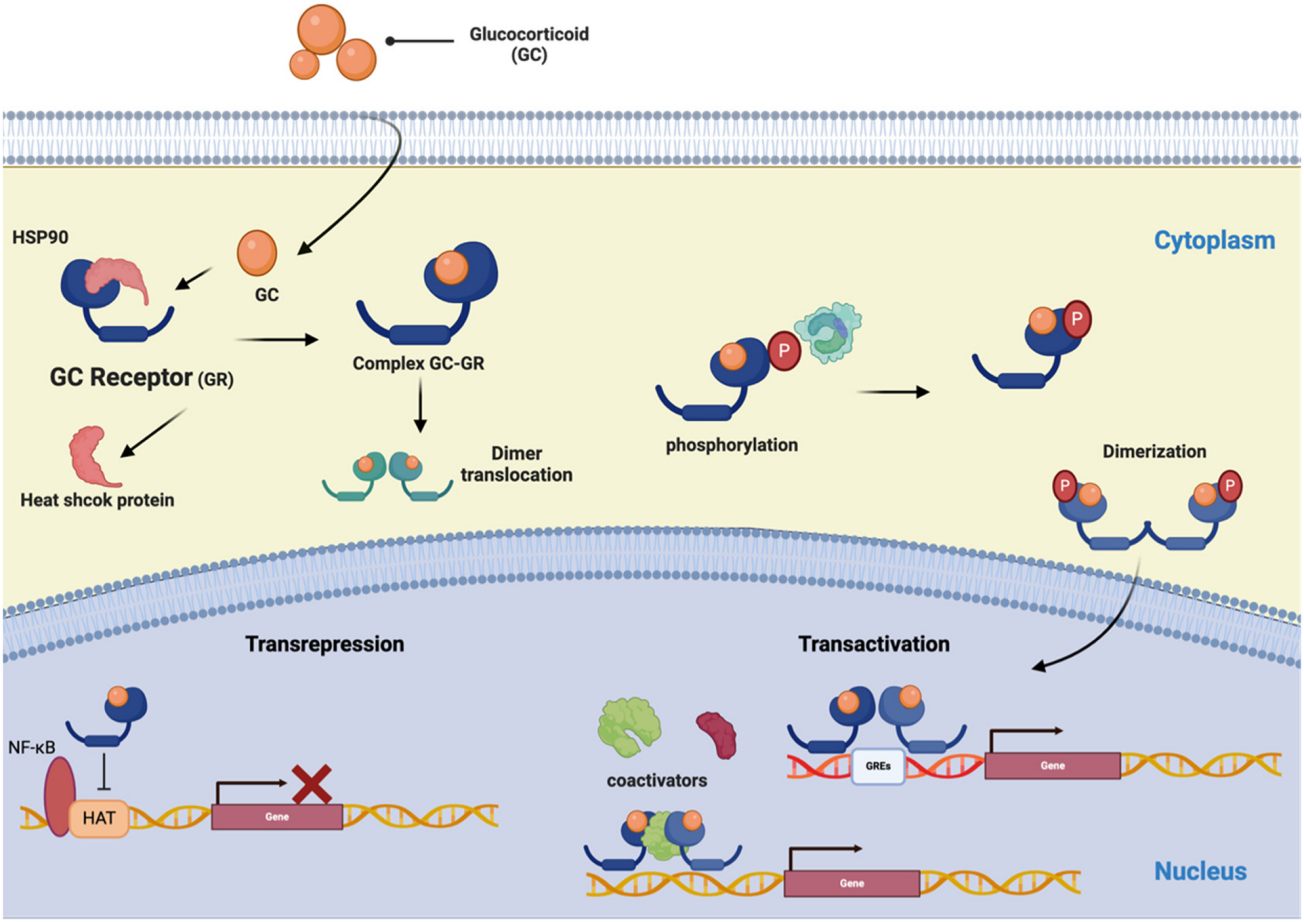
The schematic represents the GR signaling pathways. The glucocorticoid (GC) signaling pathway starts at the cell membrane and moves into the cell to bind with its receptor (GR). The receptor/corticosteroid complex can bind to the GC response element on genes coding for anti-inflammatory proteins and inhibit the synthesis of pro-inflammatory proteins by interacting with transcription factors NF-*κ*B and AP-1. (Created with BioRender).

The mechanisms by which natural or synthetic GCs mediate their genomic effects involve binding the GCs to GR homodimers in the cytoplasm, forming the GC/GR complex. This complex translocates to the nucleus, where it interacts with glucocorticoid response elements (GRE), which are 15 bp in length and consist of two pseudo-palindromic hexameric sites separated by a three bp spacer: GGAACAnnnTGTTCT (where n, is any nucleotide) (145). GRE is present in the promoter regions of steroid-sensitive genes (transactivation), or it can inhibit the activity of transcription factors (transrepression) (Figure 3) (146). Moreover, GCs have non-genomic effects that involve the direct interaction of liganded GR with diverse intracellular mediators, modulating several signaling pathways, including protein kinase C (PKC), phosphatidylinositol-specific phospholipase C (PI-PLC), and SRC kinase pathways (38).

The GR is encoded by a nine-exon *NR3C1* gene, positioned at 5q31–32 in humans. GR is a 777-amino acid multidomain protein like the other NRs; the GRs comprise the domains: a variable N-terminal domain, a C-terminal domain, and a DNA-binding domain containing zinc fingers capable of binding to DNA (122). GRs are expressed by nearly all nucleated cells, but the functional effects of glucocorticoids differ by cell type. The variety in GR signaling arises from the influence of distinct GREs and numerous receptor isoforms that emerge through alternative splicing and the commencement of alternative translations (147).

The GCs are the primary therapy for managing airway inflammation in asthma (148). The GC/GR complex leads to the expression of anti-inflammatory genes and suppression of pro-inflammatory gene expression, thus inhibiting Th2 cell-mediated inflammation in the airways. Additionally, GR interacts with coactivator molecules to suppress the expression of inflammatory genes by inhibiting the action of proinflammatory transcription factors such as NF-kB and activating protein 1 (AP-1) (149) to suppress the production and release of cytokines, proinflammatory chemokines, and airway epithelial cell adhesion molecules, which are crucial in the pathogenesis of asthma (150).

GATA3 is the master transcription factor of Th2 cells. It helps differentiate them from CD4+ lymphocytes to Th2 cells. It promotes the expression of IL-4, IL-5, and IL-13, which mediate allergic inflammation. Furthermore, it is essential for developing ILC2 progenitors (ILC2p) in the bone marrow and maintaining mature ILC2 populations in the periphery (151).

The GC/GR complex competes with GATA-3 for nuclear import via importin-α. Higher concentrations of GC increase the expression of MAP kinase phosphatase (MKP)-1, which inhibits p38 MAP kinase activity and prevents GATA-3 phosphorylation. This phosphorylation is necessary for GATA-3's interaction with importin-α and subsequent nuclear import (151). Moreover, GCs reduce the immune response and inflammation and trigger the differentiation toward Type 1 regulatory T (TR1) cells by a FOXP3-dependent mechanism (152).

## 5 Prolactin: a non-steroidal hormone

PRL is a 23 kD peptide hormone primarily produced by lactotroph cells in the anterior pituitary gland. It is named for its ability to promote lactation in response to the suckling stimulus of hungry young mammals. However, it can also be produced in the ovaries, prostate, mammary gland, brain, and immune cells (153).

Cytokines that promote PRL production, including IL-1, IL-2, and IL-6, regulate it, while endothelin-3 and IFN-γ have inhibitory effects. Post-translational modifications can result in different isoforms of PRL (153), including small, large, and macro PRL. Among these isoforms, the small one, which consists of 199 amino acids and weighs 23 kDa, is the most biologically potent (154).

PRL plays a significant role in regulating both innate and adaptive immune responses. It affects the maturation of CD4-CD8- thymocytes into CD4+ CD8+ T cells by regulating the expression of the IL-2 receptor (155). Studies suggest that PRL is involved in deregulating B cell activation, which can lead to autoimmunity (156). Moreover, PRL has been found to either promote or regulate the production of cytokines, including Th1 and Th2 type cytokines, IL-6, IFN-γ, and IL-2 (157, 158).

**Figure 4:**
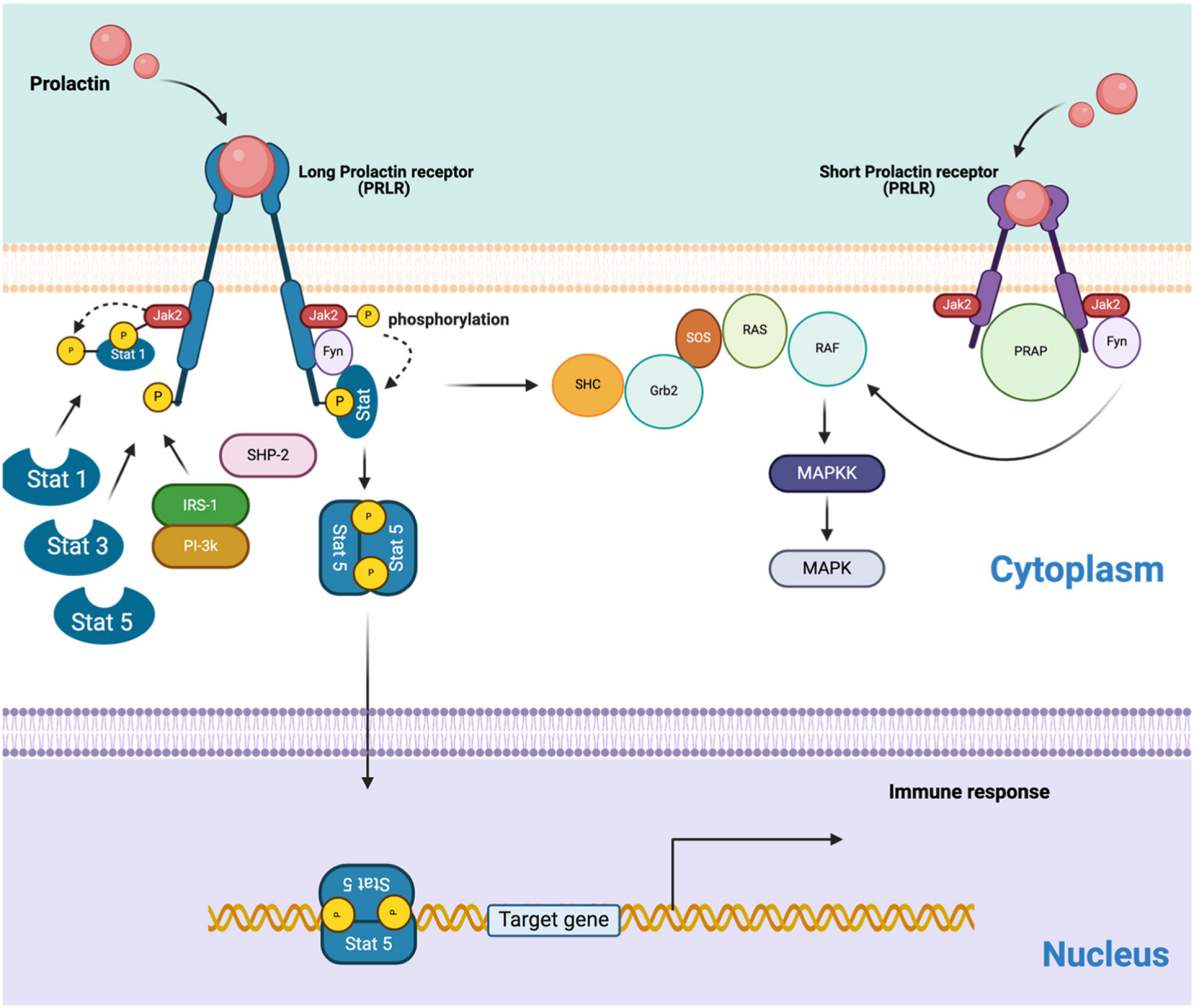
The schematic represents the PRLR signaling pathways, including both long and short isoforms. Homodimer of the hPRLR long form (LF) mediates PRL-stimulated JAK2/Stat5 signaling required for transcription/expression of PRL/PRLR target genes, which are essential for the various biological effects of the hormone. The activation of the MAP kinase pathway involves both PRLR isoforms. The connections between the JAK-Stat and MAPK pathways are also suggested, but the interactions between receptors and various transducing molecules remain unclear. (Created with BioRender).

The PRL receptor (PRLR), which belongs to the type 1 cytokine/hematopoietic superfamily, is expressed in various immune cells, including monocytes, lymphocytes, macrophages, NK cells, granulocytes, thymic epithelial cells, and Treg cells (159). Therefore, the interaction of PRL with its receptor plays a critical role in regulating immune cell proliferation, differentiation, secretion, and survival through signaling pathways (Figure 4) (160, 161).

PRL has been shown to promote the transcription of interferon regulatory factor 1 (IRF-1), a critical factor in the maturation of T and B cells. Additionally, it can trigger the production of inducible NO synthase (iNOS), an enzyme that generates NO and participates in immune responses and inflammation. Furthermore, PRL can enhance the synthesis of IFN-γ in both T and NK cells (162).

PRL is also a modulating apoptosis agent that regulates the expression of genes such as *BAX* and *BCL2*. This allows it to have an immunological maintenance function in stress states and can counteract the apoptotic effects of GCs on lymphocytes (163).

In allergies, such as eosinophilic esophagitis, PRL has been studied from the point of view of the T cells involved in inflammation: CD3+, CD4+, and CD8+, which are of great importance because they produce cytokines of the Th2 profile, PRL can regulate the production of these cytokines (164).

In addition, it has been observed that patients with allergic fungal sinusitis may present hyperprolactinemia. This rhinological disease is relatively new and is characterized by polyposis, fungal remnants, and hypersensitivity (165). Until now, the relationship of PRL with this pathology has mainly been due to the compression of the pituitary gland, which stimulates the deregulation of the synthesis of hormones (166).

On the other hand, in asthma, it has been evaluated and reported in murine models that domperidone-induced hyperprolactinemia exhibits a decrease in lymphocytes in bronchial lavage, a cellular decrease in femoral marrow lavage fluid, and mucus. Furthermore, it has been related to an increase in IL-4, IL-6, IL-10, TNF-α, and IFN-γ in the lungs, causing the allergic inflammatory response of the lungs to decrease (43).

A study conducted by Tugrul Ayanoğlu et al. compared the serum PRL levels of patients with atopic dermatitis and controls. They suggest that PRL may not play a role in disease pathogenesis (167).

Overall, while PRL's role extends beyond lactation to significant immunological functions, its exact impact on various allergic conditions remains an area of ongoing research, warranting further studies to elucidate its full spectrum of biological activities.

## 6 Hormone allergy or hypersensitivity

Paradoxically, steroid hormones can trigger what is yet a rarely diagnosed disease, which is hormonal allergy (168). Several studies have suggested that sensitization to steroid sex hormones may cause clinical symptoms such as dermatitis, dysmenorrhea, rhinitis, pruritus, and erythema multiforme (169). In severe cases of hypersensitivity to sex hormones, anaphylaxis, a life-threatening allergic reaction with rapid onset, has been observed (170). Case studies have shown that patients suffer unexplained anaphylactic reactions for years before sex hormone allergy is diagnosed correctly (171–175).

Hormone hypersensitivity can be triggered by pregnancy, exogenous hormone intake, oral contraceptives, and *in vitro* fertilization. These factors indicate multiple potential causes, including hormone administration, elevated hormone levels during pregnancy, and increased hormone sensitivity (168). The underlying mechanism remains unclear; however, it may involve IgE antibodies, T cells, an inappropriate cytokine response, natural killer (NK) cell activity, or drug hypersensitivity (176).

Progesterone hypersensitivity (PH) can occur due to natural progesterone production or after allergic sensitization to progestins used for contraception and fertility treatment, which may be recognized as foreign by the immune system (177). Thus, progestogen-specific IgE antibodies may be formed in susceptible patients following exposure to exogenous progestins. PH mainly affects young females, with fewer than 200 cases reported to date (178). As we mentioned before, one of the risk factors includes exposure to exogenous progestins and high-dose progesterone for *in vitro* fertilization. The exact cause of PH is unknown, but it is likely multifactorial due to its varied symptoms, including skin issues like dermatitis, urticaria, erythema multiforme, and other immediate reactions (179).

## 7 Discussion

Allergies are the most common worldwide diseases with an increasing prevalence and incidence. The influence of sex hormones (i.e., estrogens and androgens) on the allergic response is relevant in the appearance of autoimmune diseases and allergies in women and men (5). However, female hormones give more susceptibility to developing allergies like asthma, food allergies, or hypersensitivity than males (180). This gap in the prevalence of allergies in women and men may be regulated by hormones derived from cholesterol or peptides. Women often develop stronger immune responses, and during menstrual cycles, pregnancy, or menopause, allergic inflammation appears, perhaps because estrogens enhance the production of the Th2 cytokine profile (19, 30).

Cytokines produced by the immune system mediate the pathophysiology of allergies. About the Th2 cytokines IL-4/IL-13, some inhibitory monoclonal treatments contribute to decreasing asthma symptoms (181). However, the antibodies of the treatment show different therapeutic effects in the groups of patients, and this could be explained by the fact that some genetic alterations within the cytokines coding genes were not contemplated (182). The US Food and Drug Administration (FDA) has approved several anti-IL-5 therapies targeting eosinophilic disorders. For eosinophilic asthma, all three biologics—mepolizumab, benralizumab, and reslizumab—are approved for adults aged 18 and older, with both mepolizumab and benralizumab additionally approved for patients aged 6 and above (183).

Several research groups around the world have documented the complex connection of steroid sex hormones with menstrual cycle and allergies (168). In the case of HRT, estrogens trigger asthma symptoms due to the activation of the immune system in postmenopausal women (98). On the other hand, the treatment with Tamoxifen (estrogen receptor blocker) has reported good results in neutrophilic inflammation in animal models (184). However, its usage may cause fertility problems in the long term and some other cardiovascular side effects in both sexes (185, 186).

The use of Pg as a pro-inflammatory cytokines suppressor has shown promising results in animal models with allergic lung inflammation (114–116). However, the cases of Pg hypersensitivity are increasing because of contraceptives and fertility treatments (187). Exposure to exogenous Pg may present symptoms like dermatitis, urticaria, angioedema, asthma, or anaphylaxis. In these cases, it has been reported that the Pg desensitization protocol is effective (177, 188).

As we described above, allergic diseases are associated with low levels of testosterone and high levels of estrogen (189). In this context, replacement therapy with testosterone in men with allergies due to hypogonadism impacts positively on the anti-inflammatory cytokines stimulation and Treg differentiation (190). This replacement therapy in women with allergies has been considered, but several side effects data must be obtained before releasing a safety therapy (191).

Other hormones, like GCs, are primary immunosuppressive drugs for inflammation in asthma. However, some adverse effects are registered in patients with chronic treatments, and they are indicated only in anaphylaxis crises or emergencies associated with pro-inflammatory effects. Nevertheless, contradictory data on the severity of allergies have been shown; some studies have associated hyperprolactinemia with a decrease in asthma symptoms (43), but others have reported no differences in PRL serum levels in dermatitis (167, 192).

Hypersensitivity to hormones may include several allergic responses with different symptoms. These allergic reactions may be treated with monoclonal antibodies such as Omalizumab, which avoids anaphylaxis (193, 194).

Finally, it is essential to acknowledge the significant controversy surrounding findings on each hormone's role in either alleviating or worsening allergic symptoms. Research that considers genetic, epigenetic, and environmental factors is needed to advance the development of precision medicine tailored to individual patients. This approach will deepen our understanding of the diversity across various allergic conditions.
